# Mindfulness-based interventions for adults with ADHD: A systematic review and meta-analysis

**DOI:** 10.1097/MD.0000000000044308

**Published:** 2025-09-12

**Authors:** Hwan-Hee Kim, Nam-Hae Jung

**Affiliations:** a Department of Occupational Therapy, Semyung University, Jecheon-si, Republic of Korea; b Department of Occupational Therapy, Dongseo University, Busan, Republic of Korea.

**Keywords:** ADHD, adults, meta-analysis, mindfulness, non-pharmacological intervention

## Abstract

**Background:**

Attention-deficit/hyperactivity disorder (ADHD) frequently persists into adulthood and is associated with impairments in attention, emotional regulation, executive functioning, and quality of life. Although mindfulness-based interventions (MBIs) have been proposed as promising non-pharmacological treatments, the evidence regarding their efficacy in adults with ADHD remains inconsistent.

**Methods:**

A systematic search was conducted using the MEDLine, CINAHL, and PsycINFO databases to identify controlled trials published up to 2023. Studies were included if they evaluated the effects of MBIs in adults with ADHD using a control group, regardless of randomization. Only studies in which mindfulness was the primary therapeutic modality were included, even if limited psychoeducational or behavioral components were present. Ten studies met the inclusion criteria. Outcomes were categorized into 6 domains: self-reported and observer-rated ADHD symptoms, negative and positive affect, mindfulness skills, and functional outcomes. Meta-analyses were performed using standardized mean differences (SMDs) or mean differences with 95% confidence intervals (CIs). Risk of bias and publication bias were assessed using Cochrane tools and funnel plots, respectively.

**Results:**

Statistically significant improvements were observed in self-reported ADHD symptoms (SMD = 0.48, 95% CI [0.19, 0.76]), observer-rated ADHD symptoms (SMD = 0.32, 95% CI [0.09, 0.56]), and functional outcomes (SMD = 0.56, 95% CI [0.22, 0.90]). However, there were no significant effects on mindfulness skills (SMD = −0.20, 95% CI [−0.47, 0.08]), negative affect (SMD = 0.31, 95% CI [−0.06, 0.67]), or positive affect (SMD = −0.21, 95% CI [−0.58, 0.16]).

**Conclusion:**

MBIs may be effective in improving core ADHD symptoms and overall functioning in adults with ADHD. However, their effects on emotional well-being and mindfulness skills remain inconclusive. These findings support the utility of MBIs as complementary interventions for ADHD while highlighting the need for further high-quality studies to clarify their long-term effects and mechanisms of action.

## 1. Introduction

Attention-deficit/hyperactivity disorder (ADHD) is a neurodevelopmental disorder characterized by behavioral symptoms, such as inattention, hyperactivity, and impulsivity, typically emerging before the age of 12.^[1]^ ADHD often begins in childhood, and ~50% of individuals continue to experience symptoms into adulthood, accounting for ~2% to 4% of the adult population.^[2,3]^ In addition to behavioral symptoms, adults with ADHD frequently report low quality of life,^[4–6]^ depressive mood, low arousal, and reduced motivation (all of which are closely associated with impaired cognitive control and attentional difficulties).^[7]^

The standard treatment for ADHD generally involves pharmacological, non-pharmacological, or combined interventions, depending on age, symptom severity, and individual needs. In the United States, 30% of children and adolescents with ADHD receive medication only, 15% receive behavioral therapy only, 43% receive both treatments, and 23% receive no treatment.^[8]^ Although pharmacological treatment with central nervous system stimulants or non-stimulants has been shown to be effective in improving core ADHD symptoms such as inattention and hyperactivity/impulsivity, it is not universally effective and can cause side effects such as anxiety, agitation, and insomnia in some individuals.^[9,10]^ As a result, major clinical guidelines, including those from the CDC,^[8]^ the American Academy of Pediatrics,^[11]^ and the National Institute for Health and Care Excellence,^[12]^ recommend non-pharmacological interventions as the first-line approach when possible.

Non-pharmacological interventions for ADHD encompass a wide range of approaches, including traditional methods, such as psychotherapy, parent training, behavioral therapy, cognitive training, counseling, family therapy, dietary regulation, sensory integration therapy, working memory training, physical activity, and chiropractic care. More recently, novel interventions, such as neurofeedback, robotics-based therapy, game-based therapy, digital therapeutics, and integrated treatments, have emerged. Among these, mindfulness-based interventions (MBIs) are considered a form of cognitive training^[13–15]^ and are thought to support improvements in impaired cognitive and emotional processes in individuals with ADHD.

Mindfulness, as commonly defined, refers to intentional and nonjudgmental awareness of the present-moment experience. Bishop et al conceptualized mindfulness as “self-regulation of attention maintained on immediate experience with an attitude of curiosity, openness, and acceptance.”^[16]^ Mindfulness practices typically cultivate this quality through structured exercises such as breath awareness, body scans, or open monitoring meditation. These practices are believed to foster psychological stability by reducing emotional reactivity and disengagement from maladaptive thought patterns.^[17,18]^

MBIs primarily involve meditative practices that cultivate awareness of the present moment. However, in practice, they may also incorporate elements of psychoeducation or behavioral strategies, particularly when adapted for clinical populations, such as adults with ADHD. As a cognitive training approach, MBIs aim to improve attention, emotional self-regulation, well-being, and quality of life.^[19–21]^ Neuroimaging studies using function magnetic resonance imaging support these outcomes by demonstrating changes in brain activation patterns in regions associated with cognitive control during mindfulness states.^[20]^ Empirical evidence supports the utility of MBIs for anxiety, depression, stress-related somatic symptoms, and chronic pain^[17]^ as well as for improving symptoms in individuals with ADHD.^[13]^

While a growing number of studies have examined the effectiveness of MBIs in adults with ADHD, existing evidence remains fragmented and inconclusive.^[22,23]^ Intervention protocols vary in the degree to which mindfulness is implemented as a standalone strategy vs integrated with components such as psychoeducation or skills training, complicating direct comparisons across studies.^[24]^ Moreover, outcome measures differ widely, ranging from ADHD symptom scales to assessments of emotion regulation, quality of life, and executive functioning, making it difficult to draw unified conclusions about treatment efficacy.^[25]^

Several systematic reviews have attempted to synthesize findings in this area. For instance, Cairncross and Miller^[24]^ reported small-to-moderate effects of MBIs on attention and emotional regulation but emphasized the limited evidence for adult populations. Tercelli and Ferreira^[26]^ and Sultan et al^[27]^ focused primarily on children and adolescents, while Poissant et al^[28]^ restricted their analysis to MBIs in adults. These reviews highlight the potential of MBIs but also underscore the need for a more comprehensive and domain-specific synthesis focused on adults with ADHD.

To address these limitations, the present study conducted a systematic review and meta-analysis of randomized controlled trials (RCTs) in which MBIs were implemented as primary interventions for adults with ADHD.

## 2. Methods

### 2.1. Search strategy

A systematic literature search was conducted using the MEDLine, CINAHL, and PsycINFO databases. The search terms included “ADHD” and “mindfulness” as major keywords. Studies were included if they met the following criteria: included an experimental control group design, targeted adults diagnosed with ADHD, and implemented MBIs as the primary therapeutic component, even when accompanied by limited adjunctive strategies such as psychoeducation or behavioral skills training. Studies were excluded if mindfulness was not a central element in the intervention. This meta-analysis did not require ethical approval as it used previously published data.

### 2.2. Study selection and quality appraisal

**Figure 1.**
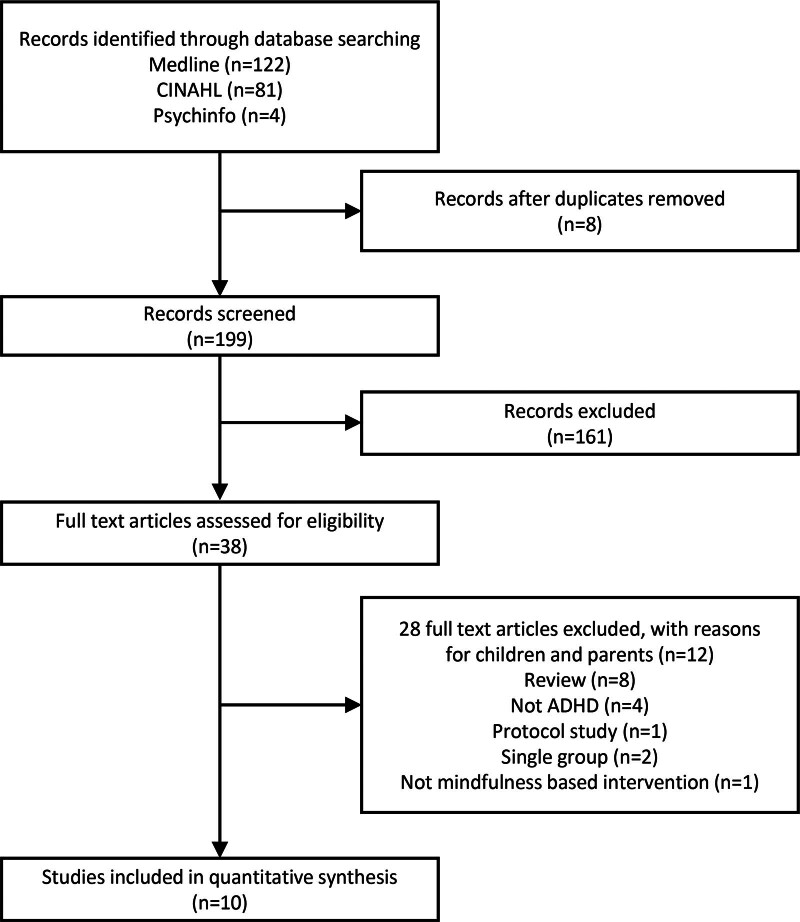
Flow diagram.

**Figure 2.**
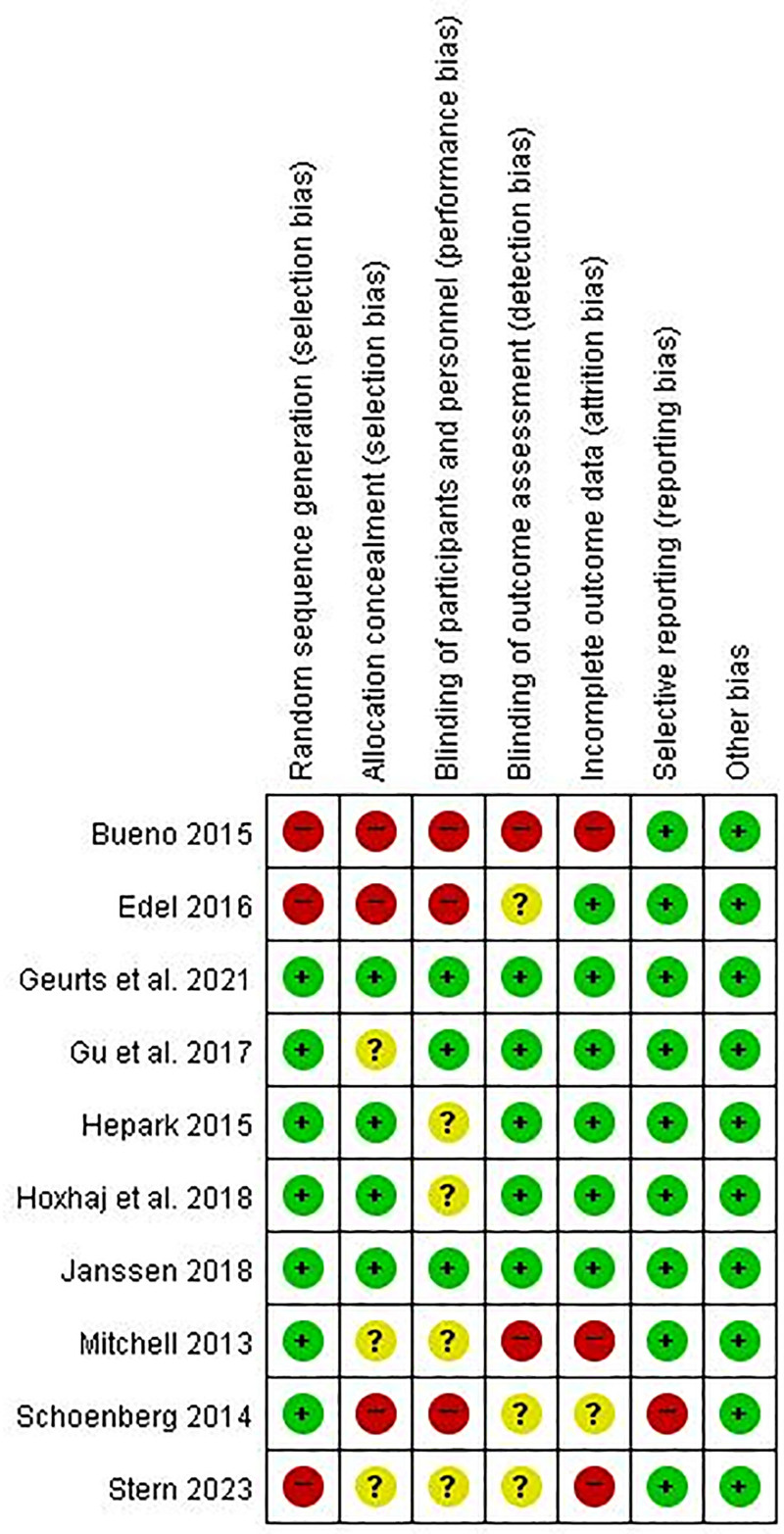
Risk of bias summary.

**Figure 3.**
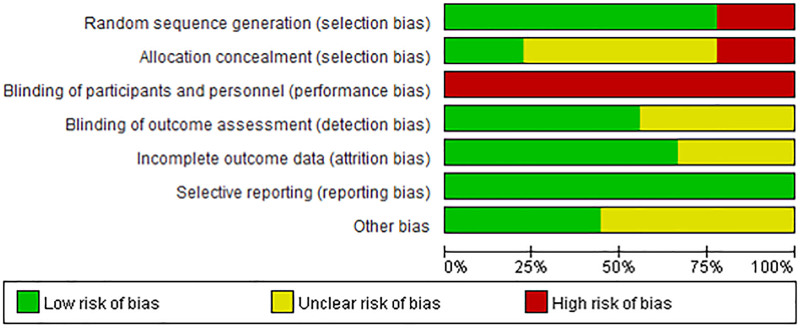
Risk of bias graph.

A total of 10 studies were included in the final analysis^[23,29–37]^ (Fig. 1). All identified studies were screened according to the PICO (Population, Intervention, Comparison, Outcome) framework. Data were qualitatively synthesized and summarized in terms of the study design, population characteristics, intervention details, outcome measures, and results. The risk of bias was assessed using the Cochrane Collaboration tool for assessing the risk of bias embedded in RevMan version 5.3 (The Cochrane Collaboration, Copenhagen, Denmark). Among the included studies, Stern et al^[34]^ were qualitatively synthesized but were excluded from the quantitative meta-analysis. Although the study met the inclusion criteria during screening, the comparison group received an active attention training intervention, making its effect size estimates noncomparable to the primary mindfulness vs passive control design included in the meta-analysis. Three independent reviewers assessed each study and disagreements were resolved through consensus discussions (Figs. 2 and 3).

### 2.3. Statistical analysis

#### 2.3.1. Heterogeneity

Statistical heterogeneity among studies was evaluated using the chi-square test and the *I*^2^ statistic. An *I*^2^ value >50% was considered indicative of substantial heterogeneity, in which case, a random-effects model was applied. When *I*^2^ was 50% or less, a fixed-effects model was used, assuming homogeneity across studies.

#### 2.3.2. Effect size

Standardized mean differences (SMDs) were calculated by dividing the difference in the means between the intervention and control groups by the pooled standard deviation. Effect sizes were interpreted following Cohen criteria: SMD ≈ 0.2 was considered small, ≈0.5 moderate, and ≥0.8 large. Meta-analysis results are presented as pooled estimates and 95% confidence intervals (CIs). Forest plots were generated to provide a visual summary of the effect sizes across the studies.

#### 2.3.3. Publication bias

**Figure 4.**
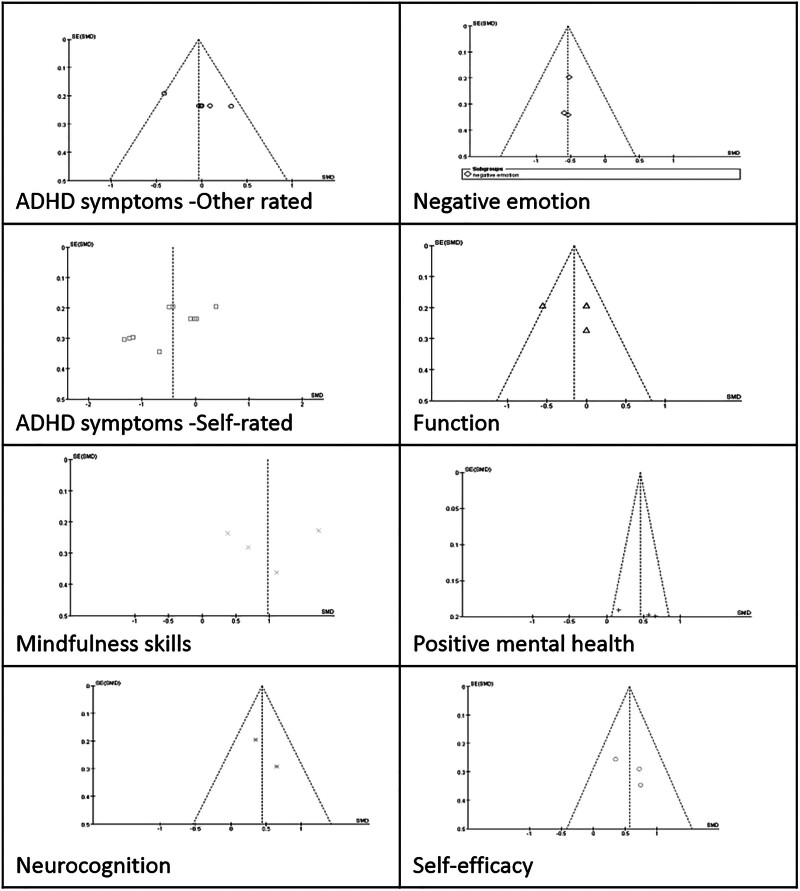
Funnel plots.

Publication bias was assessed using funnel plots, which graphically displayed the effect size (Hedges g) on the *x*-axis and the standard error on the *y*-axis. A symmetrical funnel shape indicated a low likelihood of publication bias, whereas asymmetry suggested a potential bias due to unpublished negative results or overestimated effects in smaller studies. Funnel plots were constructed for each outcome category in this meta-analysis. Visual inspection revealed symmetrical distributions centered around the pooled effect sizes, suggesting that the risk of publication bias was minimal (Fig. 4).

#### 2.3.4. Reclassification of outcome variables

To enhance consistency and comparability across studies that employed heterogeneous designs and outcome measures, reported variables were reclassified into conceptually coherent categories. This reclassification was guided by targeted theoretical domains (e.g., ADHD symptoms, emotional regulation, executive functioning) and the specific instruments used, taking into account the diversity of intervention protocols. Outcome measures were assigned to a single domain based on their primary construct and validated usage in prior literature. For example, the Five Facet Mindfulness Questionnaire (FFMQ) was categorized under mindfulness skills,^[38]^ while the Behavior Rating Inventory of Executive Function–Adult Version was mapped to the functioning domain, reflecting their focus on executive functioning in daily life.^[39]^ A complete mapping is provided in Table S1, Supplemental Digital Content, https://links.lww.com/MD/P929.

## 3. Results

### 3.1. ADHD symptoms: self-rated

**Figure 5.**
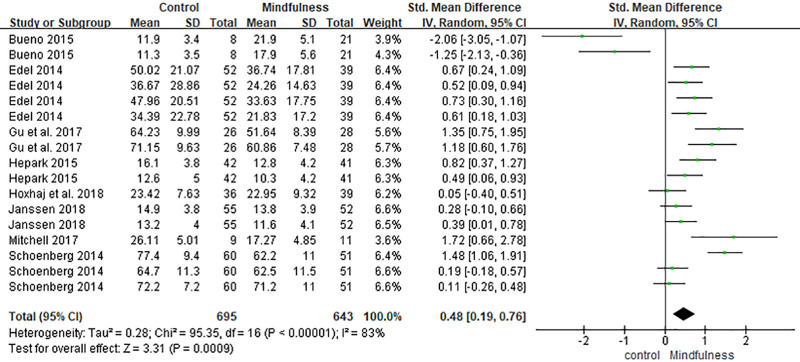
Forest plot of the effect of mindfulness-based interventions on ADHD symptoms (self-rated) in adults with ADHD. ADHD = attention-deficit/hyperactivity disorder.

Seventeen comparisons from 11 studies assessed self-rated ADHD symptoms using validated scales such as the Adult ADHD Self-Report Scale and Conners’ Adult ADHD Rating Scales-Self.^[23,29–33,35]^ A random-effects meta-analysis revealed a statistically significant moderate effect in favor of the mindfulness group (SMD = 0.48, 95% CI [0.19, 0.76], *P* = .0009). Substantial heterogeneity was observed (*I*^2^ = 83%) (Fig. 5).

### 3.2. ADHD symptoms: observer-rated

**Figure 6.**
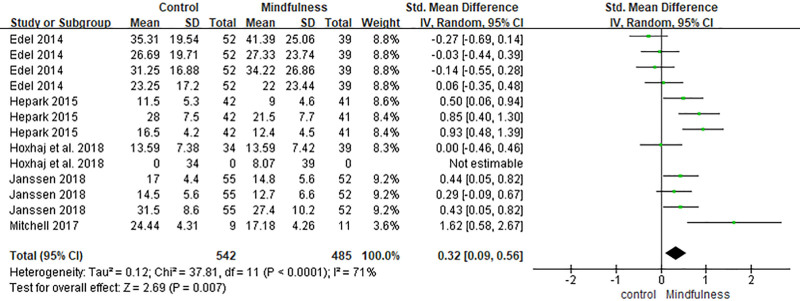
Forest plot of the effect of mindfulness-based interventions on ADHD symptoms (others-rated) in adults with ADHD. ADHD = attention-deficit/hyperactivity disorder.

Twelve effect sizes from 5 studies assessed ADHD symptoms rated by observers, with 485 participants in the intervention group and 542 in the control.^[23,30,32,35,36]^ MBIs were associated with a small but significant reduction in other-rated ADHD symptoms (SMD = 0.32, 95% CI [0.09, 0.56], *P* = .007). Moderate heterogeneity was found (*I*^2^ = 71%, *P* < .0001), suggesting variability in outcomes across studies (Fig. 6).

### 3.3. Functioning

**Figure 7.**
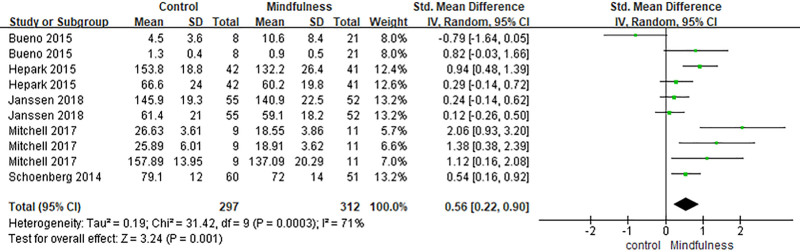
Forest plot of the effect of mindfulness-based interventions on function in adults with ADHD. ADHD = attention-deficit/hyperactivity disorder.

Ten effect sizes involving 312 participants in the intervention group and 297 in the control group examined outcomes related to general functioning.^[23,29,32,33,36]^ MBIs showed a significant positive effect on functioning (SMD = 0.56, 95% CI [0.22, 0.90], *P* = .001). Moderate heterogeneity was detected (*I*^2^ = 71%, *P* = .0003) (Fig. 7).

### 3.4. Negative emotion

**Figure 8.**
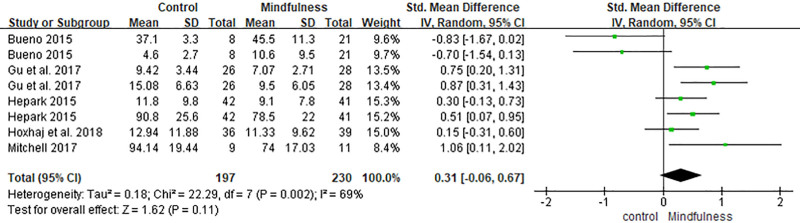
Forest plot of the effect of mindfulness-based interventions on negative emotion in adults with ADHD. ADHD = attention-deficit/hyperactivity disorder.

Eight comparisons from 5 studies evaluated negative emotional outcomes using tools such as the BDI, BAI, STAI-T, and DERS.^[30–33,36]^ Although the overall effect size favored the mindfulness intervention (SMD = 0.31, 95% CI [−0.06, 0.67]), the difference was not statistically significant (*P* = .11). Heterogeneity was moderate (*I*^2^ = 69%) (Fig. 8).

### 3.5. Positive emotion/mental health

**Figure 9.**
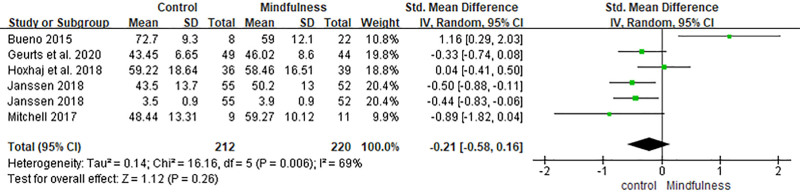
Forest plot of the effect of mindfulness-based interventions on positive mental health in adults with ADHD. ADHD = attention-deficit/hyperactivity disorder.

A total of 5 studies reported on positive affect or quality of life outcomes.^[23,33,35–37]^ The pooled SMD was −0.21 (95% CI: −0.58, 0.16, *P* = .26), indicating no statistically significant difference between mindfulness and control groups. The direction of effect slightly favored the control group, but the CI included zero. Moderate heterogeneity was observed (*I*^2^ = 69%, *P* = .006), which may be attributable to the variation in outcome measures (e.g., 36-Item Short Form Health Survey, Self-Compassion Scale-Short Form, Mental Health Continuum-Short Form) and conceptual differences across studies (Fig. 9).

### 3.6. Mindfulness skills

**Figure 10.**
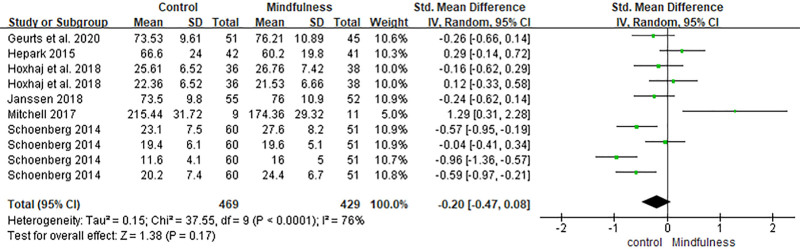
Forest plot of the effect of mindfulness-based interventions on mindfulness skills in adults with ADHD. ADHD = attention-deficit/hyperactivity disorder.

Ten comparisons from 6 studies measured mindfulness outcomes using the FFMQ, Kentucky Inventory of Mindfulness Skills, or Five Facet Act Questionnaire.^[30–32,35,36,39]^ The pooled effect size slightly favored the control group but did not reach statistical significance (SMD = −0.20, 95% CI [−0.47, 0.08], *P* = .17). Substantial heterogeneity was detected (*I*^2^ = 76%) (Fig. 10).

## 4. Discussion

This meta-analysis evaluated the multidimensional effects of MBIs in adults with ADHD. Ten studies, consisting of RCTs and quasi-experimental designs, were included. The combined sample included >600 participants across the intervention and control groups. All studies employed pre-post designs and were published between 2014 and 2023, covering diverse countries, including Germany, the Netherlands, China, the United States, and Brazil.

These findings should be interpreted with caution due to several limitations. Substantial heterogeneity was observed across multiple outcome domains, likely stemming from variation in intervention protocols (e.g., duration, delivery methods, inclusion of psychoeducational, or cognitive elements), participant characteristics (e.g., age, symptom severity, comorbidities), and the outcome measures employed. While subgroup analysis by measurement tool or intervention type could offer additional insights, the limited number of studies within each subgroup would reduce statistical power and potentially overcomplicate interpretation. Therefore, such analyses were not conducted in this study. To address conceptual and methodological variability, random-effects models were applied throughout. Future research should aim for greater standardization in intervention protocols and assessment tools to improve comparability and reduce heterogeneity.

Most interventions are based on Mindfulness-Based Cognitive Therapy or Mindfulness-Based Stress Reduction, although some incorporate elements of psychoeducation, behavioral regulation, or skills training. Although mindfulness remains a core therapeutic strategy, these adjunctive features reflect the clinical heterogeneity of MBIs. Intervention durations ranged from 6 to 13 weeks, often including homework and daily mindfulness practice. Control conditions included waitlist, treatment-as-usual, or psychoeducation. To ensure clarity regarding the intervention scope, only studies in which mindfulness was the primary therapeutic modality were included in this meta-analysis. Trials involving concurrent pharmacological treatments or combined psychotherapies were excluded during study selection. While some interventions incorporated minor psychoeducational or behavioral components, these were supportive rather than central, allowing the evaluation of MBIs as standalone treatments.

The meta-analysis revealed statistically significant positive effects of MBIs on self-rated ADHD symptoms (SMD = 4.8, 95% CI [0.19, 0.76], *P* = .0009), observer-rated ADHD symptoms (SMD = 0.32, 95% CI [0.09, 0.56], *P* = .007), and overall functioning (SMD = 0.56, 95% CI [0.22, 0.90], *P* = .001).

These findings suggest that MBIs may exert beneficial effects on both internal symptom experiences and externally observable functioning. Improvements in both symptom domains and functioning suggest that mindfulness training may support self-regulatory capacities relevant to attention, behavior organization, and role participation in adults with ADHD.^[16]^ However, further investigation is needed to clarify the underlying mechanisms, as other domains such as mindfulness skills and emotional well-being did not show statistically significant changes in this review.

One possible explanation for the lack of significant improvement in emotional well-being and mindfulness skills is the reliance on post-intervention outcomes only, rather than long-term follow-up data. Most of the included studies evaluated outcomes immediately after the intervention period, which may not capture sustained changes in trait-level mindfulness or emotional regulation. These domains often require extended practice and consolidation beyond the structured intervention phase. Future research should incorporate follow-up assessments to better determine whether MBIs yield durable improvements in emotional and dispositional outcomes.

Furthermore, while prior meta-analyses such as Oliva et al^[40]^ and Xue et al^[41]^ reported moderate-to-large benefits of MBIs across emotional and attentional domains, their scopes differed notably from the present review. Oliva et al included broader age groups and often examined adjunctive interventions, while Xue et al emphasized a mix of active and inactive controls. In contrast, the current meta-analysis focused on standalone MBIs in adults, with relatively short durations and limited active control comparisons. These methodological distinctions may account for the more conservative or nonsignificant findings in affective and dispositional domains observed in the present review.

In contrast, the analysis revealed no statistically significant effects on positive affect and quality of life (SMD = −0.21, 95% CI [−0.58, 0.16], *P* = .26), negative affect (SMD = 0.31, 95% CI [−0.06, 0.67], *P* = .11), or mindfulness skills (SMD = −0.20, 95% CI [−0.47, 0.08], *P* = .17). Notably, the direction of effect for positive affect slightly favored the control group, although the difference was not significant. While mindfulness skills showed a marginal trend toward improvement (*P* = .06), it did not reach statistical significance.

These findings suggest that while MBIs may be effective in modifying core ADHD symptoms and general functioning, improvements in emotional well-being and trait mindfulness may require longer interventions or alternative formats.

These findings are generally consistent with prior meta-analytic evidence regarding the efficacy of MBIs in ADHD populations. For example, Oliva et al^[40]^ reported that MBIs yield moderate-to-large effects on ADHD symptoms, emotional regulation, and functioning, particularly when delivered as adjunctive interventions. Our results similarly suggest that MBIs may support core symptom reduction and enhance functional outcomes, although the magnitude of effects varied depending on the domain and outcome measure. In line with Xue et al,^[41]^ we found that self-rated ADHD symptoms exhibited larger effect sizes and greater heterogeneity compared with observer-rated symptoms, highlighting the potential influence of subjective perception and expectancy on treatment outcomes. This discrepancy may be attributable to the inherently introspective nature of mindfulness practice, which may generate changes more detectable by participants themselves than by external observers.

Moreover, the lack of significant improvement in emotional well-being and mindfulness skills in the present analysis contrasts with some previous reviews that reported broader benefits of MBIs.^[42,43]^ One possible explanation is the inclusion of relatively short-duration interventions in several studies, which may have been insufficient to produce robust changes in trait-level constructs such as dispositional mindfulness or sustained emotional regulation. Another contributing factor could be the limited use of active control conditions in many trials; prior research has shown that the superiority of MBIs often diminishes when compared with active treatments, rather than waitlist or usual care controls.^[40]^

Finally, the high heterogeneity observed in multiple outcome domains underscores the need for further investigation into potential moderators. Variables such as delivery format (individual vs group), participant subtype (e.g., inattentive vs combined ADHD), and adherence to home practice may influence treatment efficacy. Future trials should aim to disentangle the specific components of MBIs that drive observed effects and determine for whom and under what conditions these interventions are most effective.

Heterogeneity levels were moderate to high in several domains. Notably, *I*^2^ was 83% for self-rated ADHD symptoms, 71% for observer-rated ADHD symptoms, and 76% for mindfulness skills. These differences may be attributed to variations in the measurement tools (e.g., Mindful Attention Awareness Scale vs FFMQ), intervention fidelity, session frequency, and participant adherence. Future research should aim to isolate the active components of MBIs and investigate whether specific formats (e.g., group vs individual delivery) or populations (e.g., inattentive vs combined-type ADHD) benefit differentially.

The risk of bias assessment showed variable quality across studies. While outcome reporting and attrition bias were generally low, many studies showed a high or unclear risk of random sequence generation, allocation concealment, and blinding procedures, particularly performance and detection bias. These risks were expected given the behavioral nature of mindfulness interventions and their reliance on self-report outcomes.

All studies included in the quantitative synthesis employed MBIs as the primary therapeutic component. One study,^[34]^ which used an active attention training as a control group, was included in the meta-analysis because the experimental group received a mindfulness-based protocol. Sensitivity analyses may be warranted to explore its influence. Its inclusion may have contributed to heterogeneity, particularly in the domains of self-reported symptoms and functioning.

Most of the included studies were conducted in Western, high-income countries, such as the United States, Germany, and the Netherlands. Cultural and healthcare system differences may influence the acceptance, implementation, and outcomes of MBIs. Future studies should examine the efficacy and adaptability of MBIs across diverse cultural, linguistic, and socioeconomic settings to improve generalizability.

Furthermore, the methodological quality of several included trials was limited by small sample sizes, inconsistent blinding, and unregistered protocols. To strengthen the evidence base, future RCTs should adopt more rigorous designs, including preregistration, adequate randomization, and standardized intervention protocols. Larger, multisite trials with longer follow-up periods are also needed to evaluate long-term effects and real-world applicability.

Overall, the findings of this meta-analysis support the clinical utility of MBIs as complementary interventions for adults with ADHD. In particular, MBIs may be effective in improving ADHD symptoms and functional outcomes (areas not always sufficiently addressed by pharmacological treatment).

High-quality future trials with dismantling designs and long-term follow-up are warranted to refine protocols and optimize implementation strategies.

## 5. Conclusion

This meta-analysis supports the effectiveness of MBIs as a complementary approach for adults with ADHD, particularly in improving ADHD symptoms and functional outcomes.

Although some trends toward improvement were observed, MBIs did not produce statistically significant changes in mindfulness skills, positive affect, or emotional well-being in this analysis. Given the observed heterogeneity across studies, future trials should adopt standardized protocols and explore contextual moderators to strengthen the evidence base.

## Author contributions

**Conceptualization:** Nam-Hae Jung.

**Data curation:** Nam-Hae Jung.

**Formal analysis:** Nam-Hae Jung.

**Investigation:** Nam-Hae Jung.

**Methodology:** Nam-Hae Jung.

**Project administration:** Hwan-Hee Kim.

**Resources:** Hwan-Hee Kim, Nam-Hae Jung.

**Software:** Hwan-Hee Kim.

**Supervision:** Hwan-Hee Kim, Nam-Hae Jung.

**Validation:** Hwan-Hee Kim, Nam-Hae Jung.

**Visualization:** Hwan-Hee Kim.

**Writing – original draft:** Hwan-Hee Kim, Nam-Hae Jung.

**Writing – review & editing:** Hwan-Hee Kim, Nam-Hae Jung.

## Supplementary Material


