# Supplementary material for: Mindfulness-based interventions for adults with ADHD: A systematic review and meta-analysis
Source: Medicine (Baltimore). 2025 Sep 12;104(37):e44308. doi: 10.1097/MD.0000000000044308 (PMC12440486; doi:10.1097/MD.0000000000044308)
Supplement: Supplementary file 1 [file medi-104-e44308-s001.docx]

| Domain | Assessment Tool | Study(ies) Using the Tool |
| --- | --- | --- |
| ADHD Symptoms – Self-rated | CAARS-S | Gu et al. (2018), Hoxhaj et al. (2018) |
| ADHD Symptoms – Self-rated | ASRS | Hepark et al. (2015), Michel et al. (2017) |
| ADHD Symptoms – Self-rated | ADHD-RS | Bueno et al. (2015) |
| ADHD Symptoms – Observer-rated | CAARS-O | Michel et al. (2017) |
| ADHD Symptoms – Observer-rated | Clinical Interview Ratings | Geurts et al. (2020) |
| Negative Emotion | DERS | Hoxhaj et al. (2018) |
| Negative Emotion | SDS | Bueno et al. (2015) |
| Negative Emotion | PSS | Gu et al. (2018), Hepark et al. (2015) |
| Positive Emotion / Mental Health | WHO-5 | Bueno et al. (2015) |
| Positive Emotion / Mental Health | PANAS-PA | Geurts et al. (2020) |
| Positive Emotion / Mental Health | SWLS | Michel et al. (2017) |
| Functioning | GAF | Geurts et al. (2020) |
| Functioning | WHODAS | Michel et al. (2017) |
| Functioning | WFIRS | Bueno et al. (2015), Hepark et al. (2015) |
| Mindfulness Skills | FFMQ | Gu et al. (2018), Hepark et al. (2015) |
| Mindfulness Skills | MAAS | Hoxhaj et al. (2018), Michel et al. (2017) |
| Neurocognition | CPT | Schoenberg et al. (2014) |
| Neurocognition | TMT | Janssen et al. (2018) |
| Neurocognition | Stroop Test | Michel et al. (2017) |
| Self-efficacy | GSES | Hoxhaj et al. (2018), Michel et al. (2017), Gu et al. (2018) |
